# Development of parallel reaction monitoring (PRM)-based quantitative proteomics applied to HER2-Positive breast cancer

**DOI:** 10.18632/oncotarget.26031

**Published:** 2018-09-18

**Authors:** Mathilde Guerin, Anthony Gonçalves, Yves Toiron, Emilie Baudelet, Matthieu Pophillat, Samuel Granjeaud, Patrick Fourquet, William Jacot, Carole Tarpin, Renaud Sabatier, Emilie Agavnian, Pascal Finetti, José Adelaide, Daniel Birnbaum, Christophe Ginestier, Emmanuelle Charafe-Jauffret, Patrice Viens, François Bertucci, Jean-Paul Borg, Luc Camoin

**Affiliations:** ^1^ Institut Paoli-Calmettes, Department of Medical Oncology, Marseille, France; ^2^ Aix-Marseille University, Inserm, CNRS, Institut Paoli-Calmettes, CRCM, Marseille Proteomics, Marseille, France; ^3^ IRCM, INSERM, Institut Régional du Cancer, Department of Medical Oncology, Montpellier, France; ^4^ Institut Paoli-Calmettes, Department of Anatomo-pathology, Marseille, France; ^5^ Aix-Marseille University, Inserm, CNRS, Institut Paoli-Calmettes, CRCM, Epithelial Stem Cells and Cancer Team, Marseille, France; ^6^ Aix-Marseille University, Inserm, CNRS, Institut Paoli-Calmettes, CRCM, Predictive Oncology Team, Marseille, France

**Keywords:** proteomics, parallel reaction monitoring, mass spectrometry, breast cancer, HER2-positive

## Abstract

**Introduction:**

treatments targeting the Human Epidermal Growth Factor Receptor 2 (HER2/ERBB2) have improved the natural history of HER2-positive breast cancer. However, except HER2 protein expression and gene amplification, there is no predictive biomarker to guide the HER2-targeted therapies. We developed Parallel reaction monitoring (PRM) a powerful approach, to quantify and evaluate key proteins involved in the HER2 pathway and/or anti-HER2 treatment sensitivity.

**Results:**

in BCLs, PRM measurements correlated with western blot immunocytochemistry and transcriptomic data. At baseline, higher expression of HER2, EGFR, PTEN and HER3 but lower expression of phospho-HER2 correlated with trastuzumab sensitivity. Under trastuzumab, PRM demonstrated a decrease in HER2 and an increase in phospho-HER2, which correlated with drug sensitivity. The opposite was observed under lapatinib. HER2 quantification was also correlated with immunohistochemistry in PDXs and clinical breast cancer samples.

**Discussion:**

in conclusion, PRM-based assay, developed to quantify proteins of the HER2 pathway in breast cancer samples revealed a large magnitude of expression, which may have relevance in terms of treatment sensitivity.

**Materials and Methods:**

we first evaluated PRM in term of sensitivity, linearity and reproducibility. PRM was then applied to breast cancer cell lines (BCLs) including BCLs exposed to anti-HER2 agents, patient-derived xenografts (PDXs) and frozen breast cancer samples.

## INTRODUCTION

During the last two decades, genomic-based approaches have shown that breast cancer is a highly heterogeneous disease with various subtypes associated with distinct prognosis and requiring different treatments [1, 2]. The growing knowledge of the molecular basis of this heterogeneity has allowed the emergence of several targeted therapies, which have begun to change the outcome of this disease. As a result it has become necessary to develop diagnostic assays to select patients’ specific therapies. Recent data emerging from whole genome sequencing approaches have shown a diversity of molecular alterations making wide range of unique diseases. This has led to the concept of personalized treatment of cancer, where more targeted therapies can be proposed but need to be selected [3].

The most famous and successful examples of targeted therapy is trastuzumab. This anti-HER2 monoclonal antibody is directed against the cell surface-expressed tyrosine kinase receptor, Human Epidermal Growth Factor Receptor 2 (HER2/ERBB2). This member of the EGFR family is amplified and overexpressed in up to 15 to 20% of breast cancers and is associated with an aggressive phenotype. Trastuzumab has transformed the natural history and prognosis of HER2-positive breast cancer patients [4–6]. However, primary or secondary resistance involvement of other HER family members, or activation of alternative survival or proliferative pathways, is almost inevitable in an advanced setting [7]. Some of these mechanisms of resistance can be potentially overcome by choosing among an increasing number of novel targeted therapies. Thus, lapatinib, a dual Epidermal Growth Factor Receptor (EGFR/HER1)/HER2 tyrosine kinase inhibitor, was approved in association with either capecitabine chemotherapy or trastuzumab itself, in patients whose disease had progressed on previous trastuzumab-based combination [8–10]. The Pan-HER irreversible inhibitor, neratinib, was recently demonstrated to improve disease-free survival when given after trastuzumab-based adjuvant treatment [11]. Additional anti-HER2 antibodies, including the antibody drug conjugates trastuzumab-emtansine, and pertuzumab, which targets another region of the extra-cellular domain of HER2, improved survival in advanced and/or trastuzumab-resistant HER2-positive breast cancer [12, 13]. The addition of pertuzumab to tratsuzumab during neoadjuvant and/or adjuvant treatment of non-metastatic breast cancer improves outcome [14, 15].

The current measurement of HER2 expression is based on immunohistochemistry (IHC) assay, a semi-quantitative approach, with positive thresholds and false positive expression, and necessitating in some cases the use of *in situ* hybridization (ISH) techniques to arbitrate on HER2 positivity and then to propose anti-HER2 treatment [16]. Moreover, IHC only allows detection of overall HER2 expression and is not able to highlight subtle changes such as post-translational modifications, which could play a critical role in cancer progression or treatment response. Finally, except the detection of HER2 protein expression and/or ERBB2 gene amplification, no current molecular assay can predict specific efficacy for a given HER2-targeted therapy.

Liquid Chromatography-Selected Reaction Monitoring (LC-SRM) is a mass spectrometry-based targeted approach, robust and of adequate sensitivity to detect and quantify thousands of fragment ions spectra of pre-specified proteins, and could ultimately replace IHC for cell lysates [17] and formalin-fixed, paraffin-embedded (FFPE) tissues [18–20]. Moreover, LC-SRM adding stable isotopic peptides (SIS) enables absolute quantification [21–24]. Parallel Reaction monitoring (PRM) is a recent alternative to SRM assays using high-resolution mass spectrometers. PRM assays are powerful targeted approaches to detect and quantify pre-specified proteins, and their activated or mutated status, in complex background and multiple samples, with a high throughput [25–28]. To our knowledge, there is currently no study that involves PRM-based analyses related to breast cancer.

We used PRM to evaluate some key proteins of the HER2 pathway to obtain a more global picture of protein expression and activation status on breast cancer cell lines (BCLs), patient-derived xenografts (PDXs) and breast cancer tumors. These assays could provide additional information to mutational and mRNA expression status of major molecular actors, eventually helping to rationalize the selection among available targeted therapies in HER2-positive-disease.

## RESULTS

### Selection and validation of proteotypic peptides for PRM-based measurements

For development of the PRM assay, multiple peptides were obtained from a trypsin digest of EGFR, HER2, HER3 and Phosphatase and tensin homolog (PTEN) on two BCLs overexpressing HER2 (BT474 and SKBR3). The best peptides of each protein, including two phospho-peptides of HER2, were then selected for quantitative targeted assays (Table 1). Selection of peptides was based on the highest number of peptide spectral match, a Mascot score greater than 30, the best intense peak area, and the absence of methionine, cysteine or trypsin missed cleavage. Initial standard curves were generated in BCLs matrix for each peptide of EGFR, HER2, PTEN and phospho-HER2 in order to identify the optimal quantification of peptides and proteins.

**Table 1:** Proteotypic peptides of EGFR, HER2, HER3, PTEN and phospho-HER2, their m/z at defined charge, the range of light and heavy peptides injected for calibration curve

PROTEIN	PEPTIDE	m/z	charge	range light	heavy	LLOD	LLOQ	CV (%)	SE
HER2	GTPTAENPEYLGLDVPV	884.4411	2	1−100 fmol	10 fmol	<1 fmol	10 fmol	2.8	0.019
	GLQSLPTHDPSPLQR	823.4365	2	1*−*100 fmol	10 fmol	<1 fmol	5 fmol	7.0	0.112
		549.2934	3	1*−*100 fmol	10 fmol	<1 fmol	5 fmol	3.1	0.045
	GIWIPDGENVK	614.322	2	1*−*100 fmol	10 fmol	<1 fmol	10 fmol	2.7	0.014
	SGGGDLTLGLEPSEEEAPR	957.458	2	1*−*100 fmol	10 fmol	<1 fmol	5 fmol	2.6	0.009
phospho HER2	GLQSLPTHDPSPLQR	863.4196	2	1*−*100 fmol	20 fmol	2 fmol	5 fmol	8.5	0.007
		575.9488	3	1*−*100 fmol	20 fmol	<1 fmol	5 fmol	12.0	0.009
	GTPTAENPEYLGLDVPV	926.4242	2	1*−*100 fmol	20 fmol	2 fmol	2 fmol	8.4	0.002
EGFR	GSTAENAEYLR	605.7886	2	1*−*100 fmol	10 fmol	<1 fmol	2 fmol	2.0	0.004
	IPLENLQIIR	604.8717	2	1*−*100 fmol	10 fmol	2 fmol	10 fmol	9.3	0.026
HER3	GVWIPEGESIK	607.8244	2	na	na	na	na	na	na
	LAEVPDLLEK	563.8213	2	na	na	na	na	na	na
PTEN	YFSPNFK	451.724	2	1*−*100 fmol	10 fmol	<1 fmol	2 fmol	6.8	0.015
	GVTIPSQR	429.2456	2	1*−*100 fmol	10 fmol	<1 fmol	2 fmol	1.8	0.003

Determination of lower limit of detection (LLOD), lower limit of quantification (LLOQ) Coefficient of Variation (CV) in percent and Standard-error (SE).

**Figure 1:**
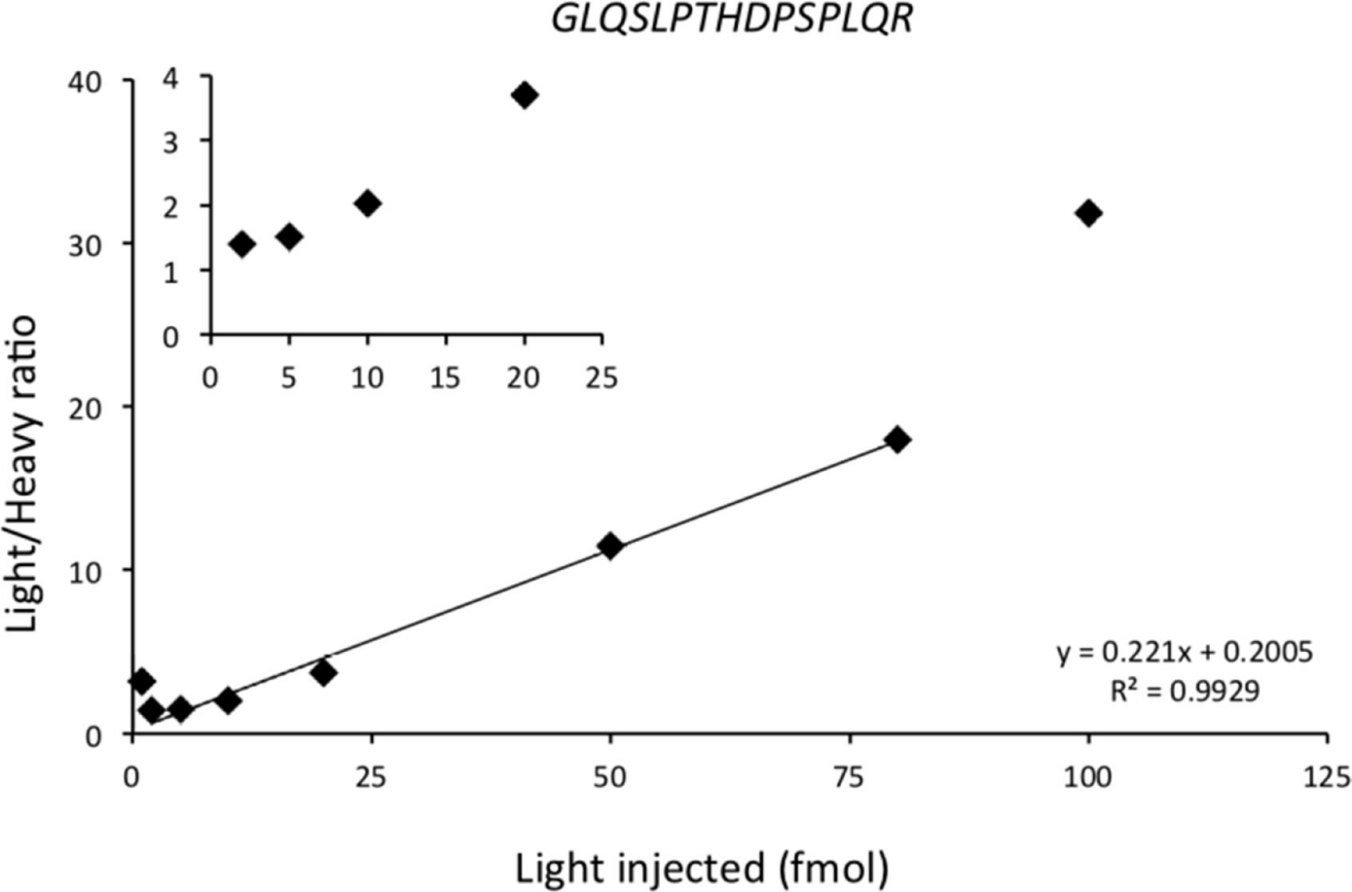
Calibration curve of GLQSLPTHDPSPLQR HER2 proteotypic peptide The calibration curve was generated using a pooled matrix of 17 BCLs. Selective bands not containing protein of interest were cut, and trypsin digested. Heavy synthetic peptide was added before injection at the concentration of 10 fmol in each sample. Light synthetic peptide was added at increasing concentrations from 1 fmol to 100 fmol. Horizontal axis represents the quantity of light synthetic peptide injected; vertical axis represents the light/heavy ratio obtained using PRM. The linearity was obtained between 2 and 80 injected fmol, with an excellent correlation (*R*^2^ = 0.99). Data are represented as mean of six technical replicates.

The calibration curve was generated from a pooled matrix of 17 BCLs, and selective bands not containing proteins of interest were cut and trypsin digested. In each standard sample, light synthetic peptide was added at increasing concentrations from 1 to 100 fmol and heavy synthetic peptide was added before injection at the concentration of 10 fmol. Lower limit of detection (LLOD) and lower limit of quantification (LLOQ) were determined as coefficient of variations (CV) and standard error (SE) at LLOQ level (Table 1). Figure 1 represents an example of calibration curve of HER2 proteotypic peptide *GLQSLPTHDPSPLQR* (for other calibration curves see Supplementary Figure 1). The calibration curve was used for each peptide to determine LLOD, LLOQ and linearity of the assay. For *GLQSLPTHDPSPLQR,* the LLOD was less than 1 fmol, the LLOQ was determined at 5 fmol, and linearity was excellent (*R*^2^ = 0.993; y = 0.221× + 0.2005). A calibration curve could not be developed for HER3 because of minute amount in the samples.

To evaluate reproducibility of this assay, we also quantified HER2 and EGFR on a protein lysate mix with different relative proportions of SKBR3 and MCF10A (an HER2-negative transformed BCL) protein extracts. The amount of HER2 and EGFR increased linearly with the increase of SKBR3/MCF10A ratio (Supplementary Figure 3).

Thus, it was possible to select proteotypic peptides from various proteins regulating the HER2-pathway and/or trastuzumab sensitivity, which could be quantitatively monitored by PRM with satisfying sensitivity, linearity and reproducibility in complex samples.

### Quantification of HER2-pathway proteins by PRM-based measurements in BCLs

#### PRM-based quantification of HER2 and correlation with immunoassays

To evaluate the concordance of PRM-measurements, with other current standard techniques, we correlated our results to HER2 immuno-assays in 17 human BCLs with a large range of HER2 expression.

**Figure 2:**
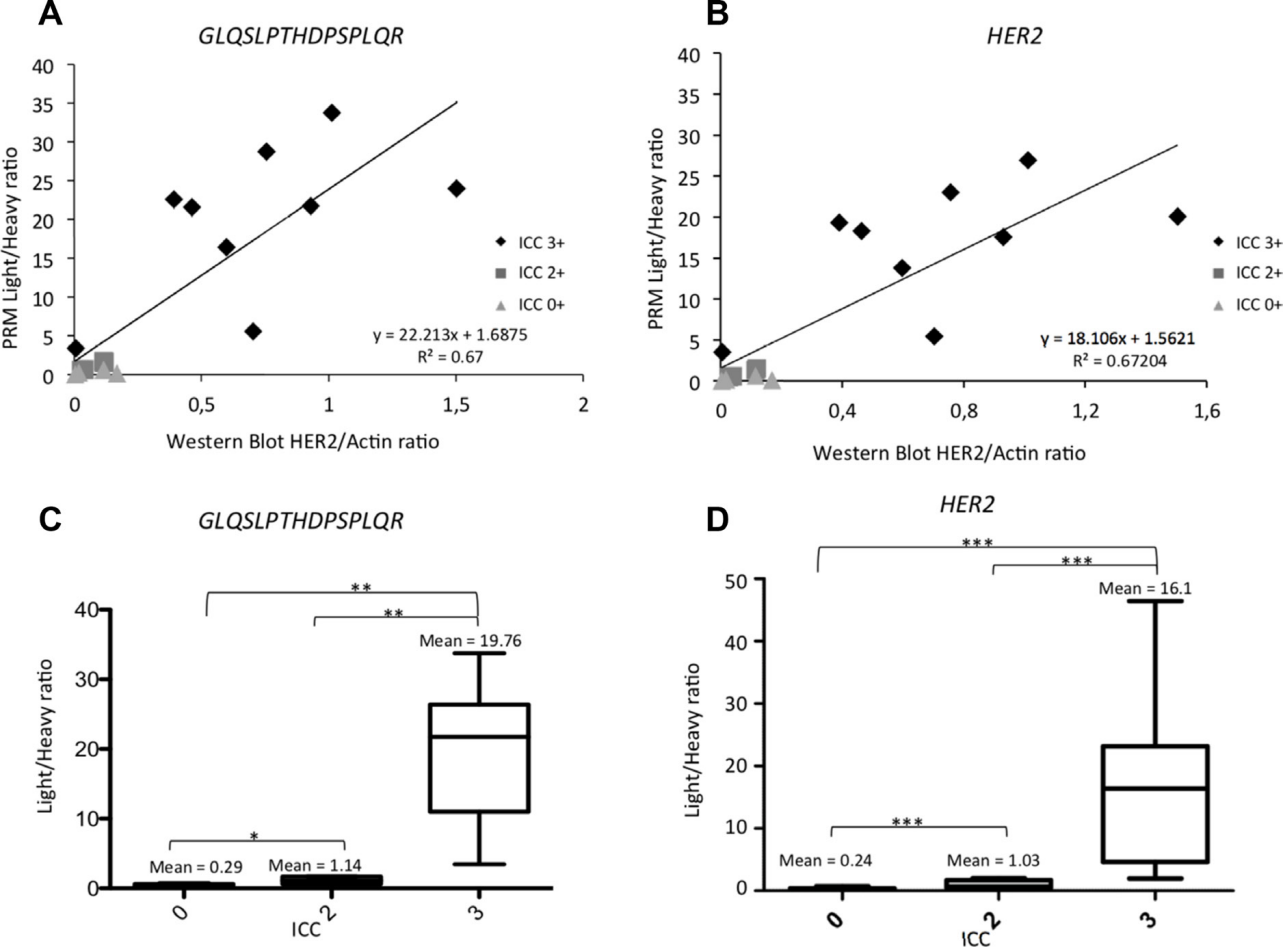
Correlation between Light/Heavy ratio obtained using PRM and Western Blot and the gold standard ICC for HER2 (**A**) Analysis of 17 BCLs. Horizontal axis represents the HER2/actin ratio obtained with western blot. Vertical axis is the light/heavy ratio of the GLQSLPTHDPSPLQR HER2 peptide of the same BCL. We also represented ICC classification of these BCL (light grey: zero expression of HER2, dark grey: equivocal in ICC; black: overexpression of HER2 in ICC. (**B**) Analysis of 17 BCLs. Horizontal axis represents the HER2/actin ratio obtained with western blot. Vertical axis is the light/heavy ratio of the mean of pooled HER2 peptides (GIWIPDGENVK; SGGGDLTLGLEPSEEEAPR; GTPTAENPEYLGLDVPV, GLQSLPTHDPSPLQR) of the same BCL. We also represented ICC classification of these BCLs (light grey: zero expression of HER2, dark grey: equivocal in ICC; black: overexpression of HER2 in ICC). (**C**) Box plot representing the light/heavy ratio of GLQSLPTHDPSPLQR peptide depending on ICC status. Significant correlation between each condition was observed: none expressors, equivocal status or overexpressors. (**D**) Box plot representing the light/heavy ratio of pooled HER2 peptides (GIWIPDGENVK; SGGGDLTLGLEPSEEEAPR; GTPTAENPEYLGLDVPV, GLQSLPTHDPSPLQR) depending on ICC status. Significant correlation between each condition: ns = non significant; ^*^p < 0.05; ^**^p < 0.01; ^***^p < 0.001. Data are represented as mean of six technical replicates ± standard deviation.

Proteins of interest were measured using PRM and their relative quantification was compared to western blot. A good correlation was found with an average *R*^2^ factor between light/heavy peptide ratio by PRM of 0.67 for HER2 peptides. For example, the R^2^ factor was 0.67 for *GLQSLPTHDPSPLQR* between light/heavy peptide from PRM and western blot (Figure 2A and 2B). Similar results were obtained with the other HER2 peptides (see Supplementary Figure 2A–2C).

Four of the 17 BCLs were negative (0–1+) by immunocytochemistry (ICC), four had equivocal status (2+) and nine were positive (3+). All BCLs with 0/1+ or 2+ with ICC had small amounts of HER2 using PRM (under the median). All HER2-positive BCLs (3+) had HER2 PRM values above the median. The means of light/heavy ratio obtained for HER2 were 0.24, 1.03 and 16.1 for negative, equivocal and positive BCLs, respectively, with a difference between each group (*p* < 0.001) (Figure 2C and 2D and Supplementary Figure 2D–2F).

Thus, PRM-based relative quantification of HER2 expression correlated well with western blot and ICC scores in human BCLs.

#### PRM-based absolute quantification of HER2-pathway proteins

We next used PRM-based analysis to provide an absolute quantification of HER2, EGFR, and PTEN proteins, in addition to two HER2 phospho-peptides and a relative quantification of HER3 (Supplementary Table 2).

**Figure 3:**
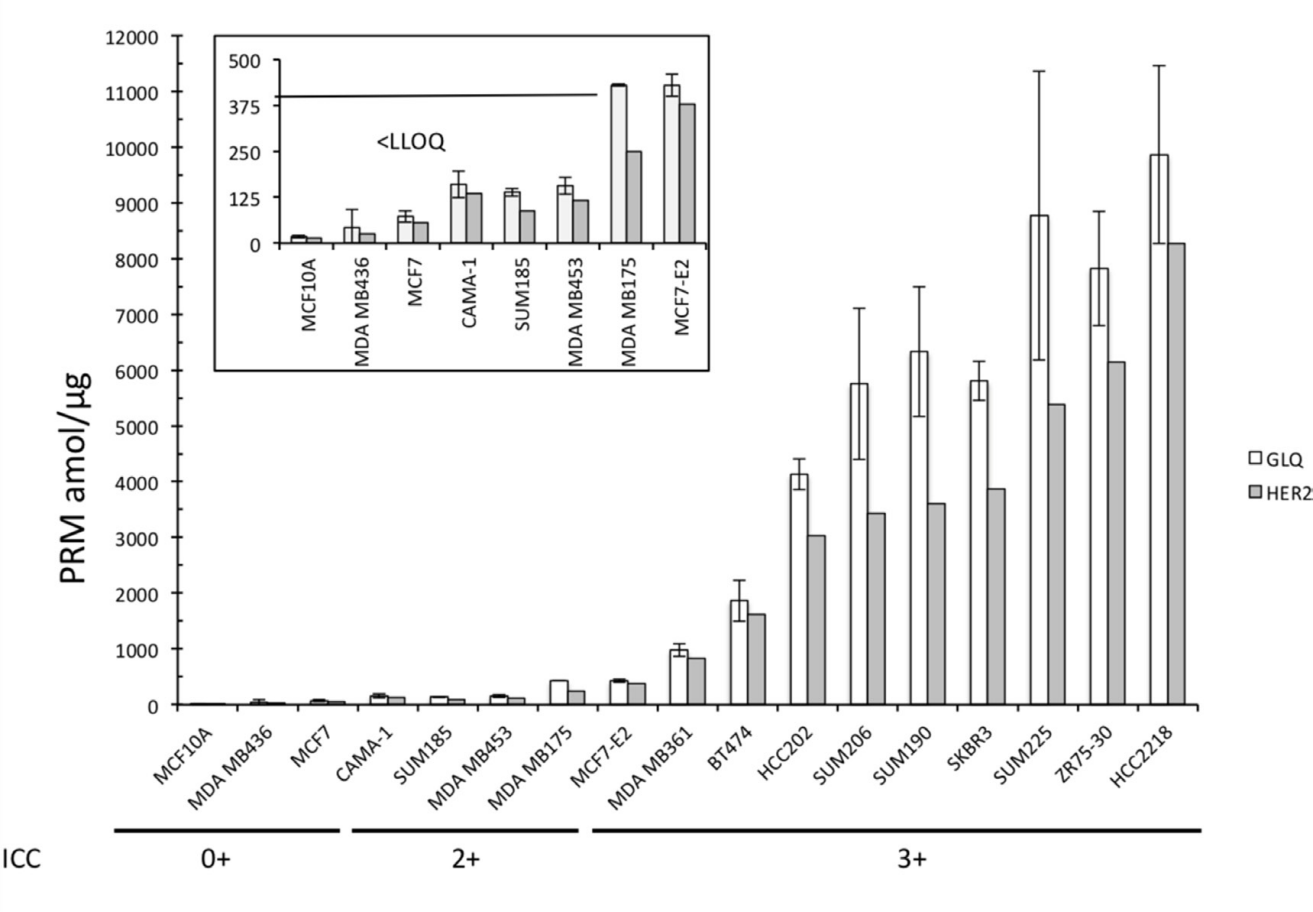
Absolute quantification of GLQSLPTHDPSPLQR and the pooled HER2 peptides of the 17 BCLs Box Plot representing the large magnitude of range of HER2 expression, from under LLOQ to 9860 amol/µg. Data are represented as mean of six technical replicates ± SEM.

All the four HER2-negative BCLs presented no quantification of the HER2 peptide *GLQSLPTHDPSPLQR* (value under LLOQ) (Figure 3).

Conversely, the range of quantification was large across the 13 HER2-positive BCLs: under LLOQ to 9860 amol/µg for *GLQSLPTHDPSPLQR* peptide. The four BCLs annotated 2+ by ICC were found between under LLOQ (SUM185 and MDAMB453) and 430 amol/µg (MDAMB175 and MCF-E2), whereas ranged from 978 (MDA-MB361) to 9860 amol/µg (HCC-2218) for BCLs annotated 3+ by ICC (Supplementary Table 2 and Figure 3).

These results highlighted the wide magnitude of expression range in HER2-positive BCLs and the potential of absolute quantification techniques compared to semi-quantitative approaches such as ICC.

#### Quantitative variations of HER2-pathway proteins under anti-HER2 treatment

Five HER2-positive BCLs (BT474, SKBR3, SUM190, SUM225 and ZR75-30) were treated with trastuzumab or lapatinib.

**Figure 4:**
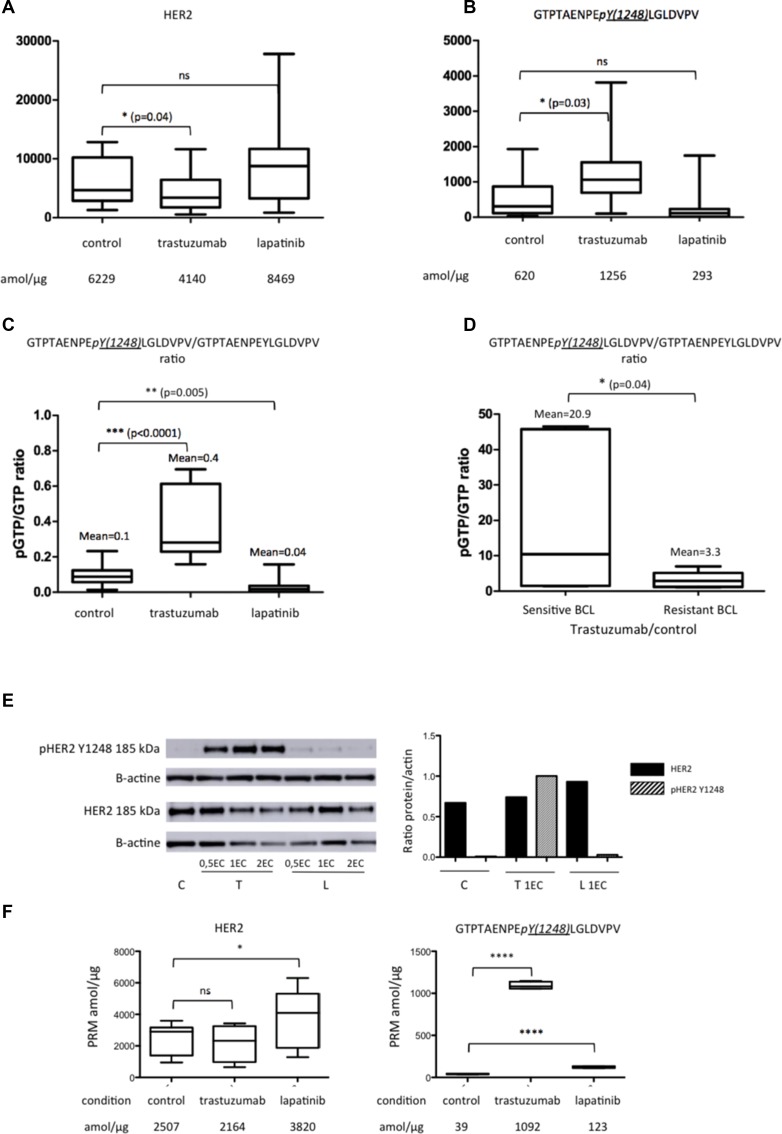
Expression of HER2 peptides on the five BCLs (BT474, SKBR3, SUM190, SUM225 and ZR75-30) in control (C) condition and under trastuzumab (T) or lapatinib (L) treatment (**A**) represents the mean of the pooled non-phosphorylated peptides (GIWIPDGENVK; SGGGDLTLGLEPSEEEAPR; GTPTAENPEYLGLDVPV, GLQSLPTHDPSPLQR); (**B**) the phosphorylated HER2 peptide GTPTAENPEpY(1248)LGLDVPV; (**C**) the mean of GTPTAENPEpY(1248)LGLDVPV/GTPTAENPEYLGLDVPV ratio; (**D**) the mean of GTPTAENPEpY(1248)LGLDVPV/GTPTAENPEYLGLDVPV ratio under trastuzumab compared to control condition in sensitive and resistant BCL, determined by Ginestier *et al.*; (**E**) western blots of HER2 and phospho-HER2 peptide GTPTAENPEpY(1248)LGLDVPV on SKBR3 breast cell line; (**F**) the mean of the pooled non-phosphorylated peptides (GIWIPDGENVK; SGGGDLTLGLEPSEEEAPR; GTPTAENPEYLGLDVPV, GLQSLPTHDPSPLQR) and phospho-HER2 peptide GTPTAENPEpY(1248)LGLDVPV on SKBR3 breast cell line. Significant correlation between each condition: ns = non significant; ^*^p < 0.05; ^**^p < 0.01; ^***^p < 0.001; ^****^p < 0.0001. EC = Effective Concentration. PRM data are represented as mean of six technical replicates ± SEM.

When BCLs were treated with lapatinib, we observed a non-significant increase in the amount of HER2 and a non-significant decrease in the amount of HER2-phosphorylated peptide (Y1248) *GTPTAENPEYLGLDVPV*, compared to control conditions (Figure 4A and 4B). However, when we quantified the phosphorylated part of *GTPTAENPEYLGLDVPV* compared to its non-phosphorylated counterpart (*pGTPTAENPEYLGLDVPV*/*GTPTAENPEYLGLDVPV ratio)*, the ratio decreased significantly under lapatinib treatment (mean = 0.04 *vs*. 0.1; *p* = 0.005) (Figure 4C). Regarding the other HER2-pathway proteins EGFR, HER3 and PTEN, only the amount of EGFR was found significantly higher compared to control condition (*p* = 0.02) with large standard error of the mean (SEM) (see Supplementary Figure 4).

Under trastuzumab, the reverse phenomenon was observed: the amount of HER2 significantly decreased, whereas the amount of phosphorylated peptide (Y1248) *GTPTAENPEYLGLDVPV* increased significantly (Figure 4A and 4B). The mean of *pGTPTAENPEYLGLDVPV*/*GTPTAENPEYLGLDVPV* ratio was 4 times higher than in control condition (Figure 4C). This ratio was higher on sensitive BCLs (BT474, SKBR3 and ZR75-30) compared to resistant BCLs (SUM190, SUM225) (Figure 4D). The increase of the amount of phosphorylated peptide (Y1248) GTPTAENPEYLGLDVPV was of particular significance for the SKBR3 BCL (Figure 4E) and was confirmed on western blot analyses. No significant difference was observed for EGFR, HER3 and PTEN (see Supplementary Figure 4).

Thus, PRM-based protein quantification was able to detect pharmacodynamic variation, including phospho-peptides that could provide useful information for treatment sensitivity.

#### Quantification of HER2-pathway proteins and trastuzumab resistance

**Figure 5:**
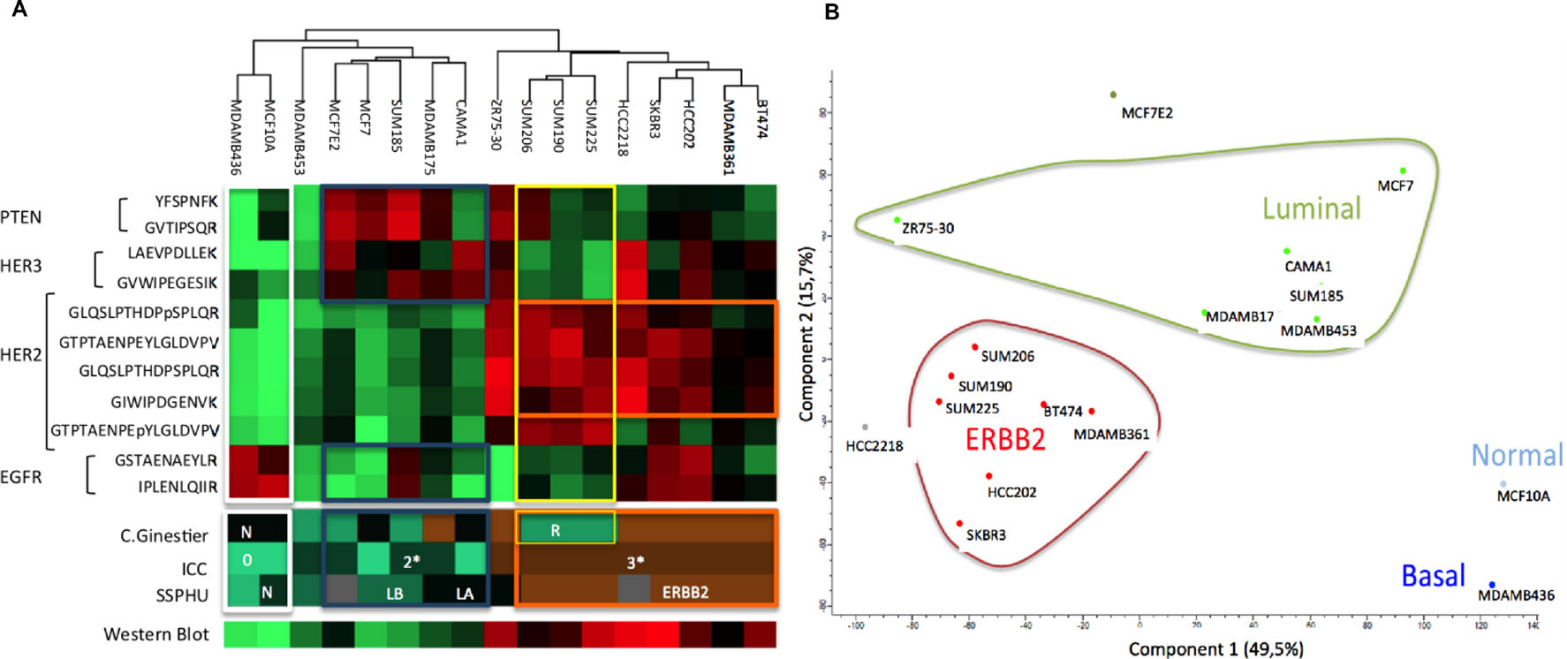
Protein expression of breast cell lines for EGFR, HER2, HER3 and PTEN and the corresponding proteomic classifications of Ginestier, ICC, HER2 expression obtained using western blot and transcriptomic classification SSPHU HeatMap (**A**) and graphic representations of Principal Component analysis (**B**).

We next examined the correlation between trastuzumab sensitivity of the various BCLs and expression of HER2-pathway proteins. Figure 5A represents the relative expression of proteotypic peptides of EGFR, HER2, HER3, PTEN, and phospho-HER2 on the 17 BCLs, depicted as a heatmap of hierarchical clustering.

For the HER2 subgroup, two patterns were identified: the first, with a higher amount of PTEN, EGFR, and HER3, but a small amount of phosphorylated HER2 peptide (Y1248) *GTPTAENPEYLGLDVPV*. All these BCLs had been classified « sensitive » by Ginestier classification, based on the measure of cell viability by cell titer under trastuzumab treatment [25].

The « resistant » subgroup conversely expressed phosphorylated peptide (Y1248) *GTPTAENPEYLGLDVPV* but had a lower quantity of HER2, PTEN, EGFR, and HER3. Most of the sensitive BCLs (6 of 7) had a higher amount of HER2 (>500 amol/µg for *GLQSLPTHDPSPLQR*) compared to only 3 of the 6 resistant BCLs.

Thus, protein expression of molecular actors from the HER2-pathway, as determined by PRM, could be associated with trastuzumab sensitivity.

#### PRM-based measurements of HER2-pathway proteins and previously defined transcriptomic signatures

We previously used the single sample predictor (SSP) classification [29] to determine a basal/luminal signature applied on BCLs. We evaluated the correlation between this genomic classification applied to BCLs and PRM-based protein expression. Figure 5A is a HeatMap representing all the 17 BCLs clustered depending on the expression of HER2, EGFR, HER3, PTEN, and phosphorylated part of HER2 using PRM.

We compared the trained clusters to the genomic SSP classification depending on HER2 expression. For most BCLs, those classified as “ERBB2” by SSP presented an overexpression of HER2 at the protein level using PRM (outlined in orange) (SUM206, SUM225, HCC2218, SKBR3, MDAMB361 and BT474). All these BCLs were 3+ by ICC and had high expression of HER2 by western blot. Conversely HER2-negative “normal”-SSP MCF10A cell line and most of the “luminal”-SSP BCLs (MCF7, SUM185, MDAMB175, CAMA1, MDAMB436, and MDAMB453) had low levels of HER2. These BCLs were classified 2 + or zero using ICC and showed a low expression of HER2 in western blot. We also observed that a majority of “ERBB2”-SSP BCLs did not express PTEN, while the “luminal”-SSP BCLs expressed PTEN and HER3 but not EGFR. Principal component analysis of the protein dataset showed a clear arrangement according to the SSP classification (Figure 5B).

Thus, protein-PRM measurements of molecular actors from the HER2-pathway correlated with previously defined transcriptomic signatures.

### Quantification of HER2-pathway proteins by PRM-based measurements in breast cancer patient samples

**Figure 6:**
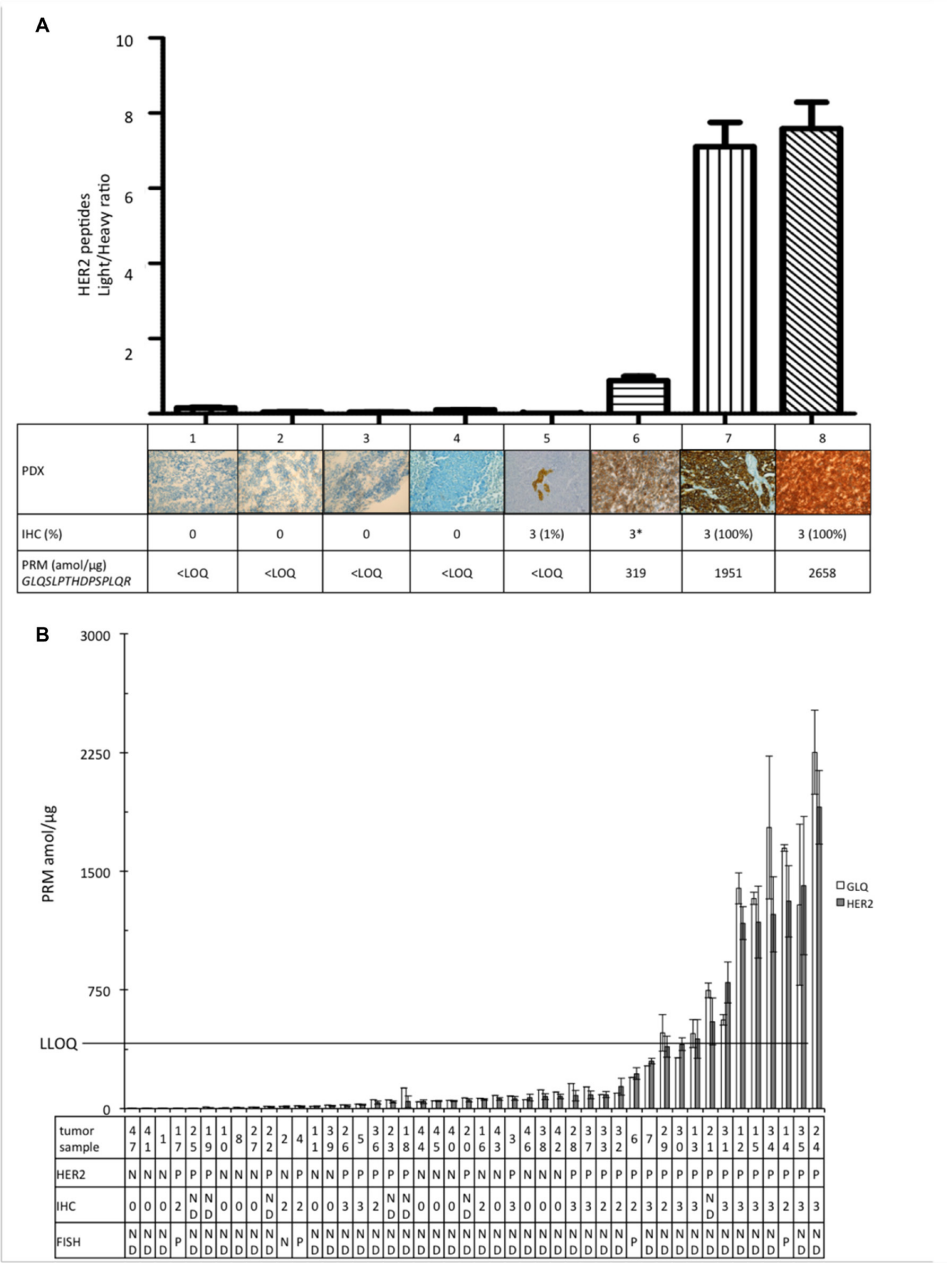
Quantification of the pooled HER2 peptides (GIWIPDGENVK; SGGGDLTLGLEPSEEEAPR; GTPTAENPEYLGLDVPV, GLQSLPTHDPSPLQR) (**A**) Light/heavy ratio of the 8 PDX. Each image represents the score corresponding HER2 expression on IHC. ^*^Sample 6 was classified 3+ on IHC of PDX as it was 2+ on IHC of corresponding patient. (**B**) HER2 absolute quantification of the 46 tumor samples. The table represents the corresponding characteristics of the tumors: HER2 defined with IHC: N = negative; P = positive; ND = not determined (0 = 0 cross negative status; 2 = 2+ equivocal status; 3 = 3+ positive status) and/or with FISH. LLOQ line represents the limit of absolute quantification. Data are represented as mean of four replicates ± standard deviation.

PRM-based assay performance was then studied by analyzing tumor tissues obtained from patients. Eight PDXs from human breast cancers were examined and showed a good agreement between PRM-based measurements and IHC for HER2 quantification (Figure 6A). Three magnitude ranges of HER2 expression: negative PDXs (samples 1 to 5) had PRM quantification of *GLQSLPTHDPSPLQR* under LLOQ; samples 7 and 8, IHC 3+, had PRM values around 2000 amol/µg. To note, sample 6 was classified 3+ on the PDX sample, but 2+ on the corresponding patient tumor and *GLQSLPTHDPSPLQR* quantification was found lower (319 amol/μg) than in other HER2-positive PDXs.

The ability of the PRM assay to detect and quantify the selected proteins of interest and to determine different profiles of protein expression were further evaluated in 46 frozen breast cancer samples (Figure 6B). Breast cancer samples had various profiles of aggressiveness and came from various sources as mentioned in Methods. All HER2-negative tumors had *GLQSLPTHDPSPLQR* amount under the LLOQ and all tumors with *GLQSLPTHDPSPLQR* higher than LLOQ were classified HER-2 positive by IHC. However, some tumors were classified as positive using IHC but had only a small amount of HER2 assessed by PRM assay. We then analyzed EGFR, HER3, and PTEN in HER2-positive samples, as determined using the gold standard IHC. These proteins were in small amount and EGFR and PTEN could only be quantified in a few samples (see Supplementary Figure 5). There was no correlation between HER2 level and EGFR, HER3 or PTEN amount. The three tumors expressing phosphorylated peptide (Y1248) *GTPTAENPEYLGLDVPV* had high levels of HER2.

Because most of the patients had localized tumors, they were treated with surgery and were in complete remission after adjuvant therapy including trastuzumab, median disease-free survival (DFS) and overall survival (OS) were not reached, and hence correlation between PRM level of HER2 and survival could not be established.

Nevertheless, we observed survivals on the twenty-nine HER2-positive breast tumors: out of the twenty HER2-positive and low PRM HER2 group (<405 amol/μg, corresponding to the mean PRM values of the HER2-positive breast samples), six patients relapsed or progressed, whereas only one progression or relapse event was observed among the nine high-PRM HER2 expressing group (>405 amol/μg).

Among the nine patients with high PRM level of HER2 expression, no death was observed whereas four patients of the twenty with low PRM level of HER2 died due to cancer progression.

Among the twenty early breast cancers, seven had high level of PRM-based HER2 compared to two out of nine advanced (inflammatory or metastatic) breast cancer, with no difference *(p = 0.35)*.

There was also no difference of HER2 expression depending on Hormone Receptors (HR) expression.

## DISCUSSION

*ERBB2* amplification (and the resulting protein overexpression) has been identified as a major oncogenic driver in a subset of breast cancers, and its targeted therapeutic modulation by trastuzumab has become a paradigm for successful precision medicine in oncology. Other anti-HER2 therapies have emerged, which can be combined with trastuzumab, either concomitantly or sequentially after trastuzumab failure. However no effective biomarker other than HER2 expression by IHC and/or amplification by ISH, is considered when selecting available therapies. In addition, a number of patients experience treatment failure, but no biomarker is able to predict clinical outcome in patients receiving anti-HER2 treatments.

Current determination of HER2 clinical status is based on IHC, a semi-quantitative approach with positive thresholds and false-positive expressors. Therefore, there is a crucial need to develop more sensitive and specific assays to detect and quantify HER2, as well as other proteins implicated in the HER2 pathway. LC-SRM and LC-PRM assays are sensitive and robust mass spectrometry-based targeted approaches, which that detect and quantify thousands of fragment ions spectra of pre-specified proteins. Moreover, time and cost for LC-PRM assays are at present only slightly higher than IHC assays and should be improved by the development of faster and more robust, next-generation spectrometers allowing large multiplexing. Therefore, LC-PRM assays have the potential to ultimately replace IHC.

We have developed an LC-PRM assay to detect and quantify various proteins implicated in the HER2 pathway. This provides a more global picture of protein expression and activation, which complements current IHC/ISH-based standard approaches and might guide in the choice of the different available targeted therapies.

We first selected the most suitable proteotypic peptides from HER2, phospho-HER2, EGFR, HER3 and PTEN for the PRM assay. Standard curves were generated by using BCL matrix and adding SIS-peptides in known concentration. LLOD and LLOQ were defined and linearity and reproducibility demonstrated for all the selected peptides, except for HER3 due to its low concentration in samples. PRM-based quantification of HER2 expression correlated with western blot quantification for BCLs and with ICC/IHC expression for BCLs, PDXs and frozen breast cancer samples. As an example in the latter case, all HER2-negative samples by IHC had a low amount of HER2 protein as assessed by PRM, whereas all samples with high level of HER2 by PRM were classified as HER2-positive by IHC or Fluorescent *in situ* hybridization (FISH).

The PRM-based assay revealed a large dynamic range of HER2 concentrations (0 to 1694 amol/µg) in breast cancer samples, all considered as HER2-positive by IHC. Also it was of note that a significant number of tumors were classified as positive with gold standard IHC and/or FISH but had low amount of HER2 using PRM (under LLOQ). Such heterogeneity in HER2-positive BCLs as well as in FFPE tissues of HER2-positive breast cancers has been reported [19]. Many HER2-positive tumors with 3+ IHC did not express elevated HER2 protein level by SRM-based assessment, suggesting that these patients may not benefit from trastuzumab treatment.

PRM-based HER2 protein quantification also correlated with HER2 status as defined by SSP, a transcriptomic classification specifically dedicated to BCLs: most of BCLs classified as ”ERBB2” by SSP had a high amount of HER2 protein using PRM, whereas “basal” and “luminal”-SSP BCLs had a low amount of HER2 protein. However, as also described by the Cancer Genome Atlas Network in 2012, there could be some discrepancy between HER2 genomic or transcriptomic analyses and proteomic data [2]. Thus, in our study, ZR75-30 BCL was classified as “luminal” by SSP classification, although it expressed a high amount of HER2 protein.

We have also examined how PRM-based assessment of HER2 and HER2-pathway related proteins may inform on response to anti-HER2 therapeutics. We first observed that most of trastuzumab-sensitive HER2-positive BCLs (6/7) had a high amount of HER2, while half of resistant BCLs (3/6) had a low level of HER2. Interestingly, most of trastuzumab-sensitive BCLs also expressed EGFR, HER3, and PTEN, but had a low level of phospho-HER2. Thus, beyond HER2 levels, phospho-HER2 and its variation under treatment, as well as baseline expression of other actors involved in HER2 pathway such as PTEN, EGFR and HER3, may better inform on trastuzumab sensitivity.

We then examined quantitative variations of HER2, phospho-HER2 peptides, EGFR, HER3 and PTEN, under trastuzumab and lapatinib treatment in HER2 positive BCLs. Compared to control condition, phosphorylated HER2 peptide (Y1248) *GTPTAENPEYLGLDVPV* increased under trastuzumab and decreased under lapatinib exposures. Sensitive BCLs had a higher increase of p*GTPTAENPEYLGLDVP*/*GTPTAENPEYLGLDVP* ratio under trastuzumab (mean = 20.9), compared to resistant BCLs (mean = 3.3). We previously evaluated by ICC the phosphorylation status of Y1248 located in the peptide *GTPTAENPEYLGLDVPV* before and after trastuzumab exposure in HER2-positive BCLs [26]. For sensitive BCLs such as BT474 or ZR75-30, more than 50% cells were positive for phosphorylation of Y1248, whereas in another sensitive cell line such as SKBR3 10 to 50% of cells were positive. Y1248 was phosphorylated in less than 10% of cells in resistant BCLs. In addition, after treatment with trastuzumab, less than 10% cells expressed phosphorylated Y1248. These results are in contradiction with our PRM-based quantification of phosphorylated peptide (Y1248) GTPTAENPEYLGLDVPV. The reason for this discrepancy is not clear, but ICC only measures the membrane part of the peptides and does not represent the ideal assay for phosphorylated cytoplasmic peptides. Instead, PRM allows the quantification of all phosphorylated peptides and is therefore more precise for quantifying the global Y1248 phosphorylation. It is important to note that our results were coherent with those from western blot, suggesting a better quantification than IHC (Figure 4E and 4F). By studying the mechanism of action of trastuzumab and lapatinib on SKBR3 and MCF7-HER2 BCLs, it has been shown that lapatinib, a small tyrosine kinase inhibitor targeting both HER2 and EGFR, prevents HER2 ubiquitination and degradation, explaining the accumulation of inactive HER2 and EGFR on cytoplasmic membrane [30]. Trastuzumab has been described to have opposite effects, increasing ubiquitination and degradation of the receptor with an increase of the phosphorylated HER2 part [30]. Phosphorylation of Y1248 may have a negative regulatory effect, inhibiting AKT phosphorylation and could therefore mediate growth inhibition [31]. Our data are consistent with these hypotheses and illustrates how dynamic and quantitative protein analysis may contribute to explain mechanisms of action of drugs as well as predicting treatment efficacy.

We were interested to test whether PRM-based quantitative measurements of HER2-pathway proteins can predict prognosis, but we were not able to evaluate a survival correlation in our study. Indeed, most analyzed patients had early breast cancer tumors, treated by optimal local treatment and adjuvant trastuzumab-based treatment and did not relapse at this time. A correlation between high HER2 level and increased survival in breast patients treated with anti-HER2 therapy has been suggested [32] using SRM-MS approach and quantification of HER2 protein levels in 270 FFPE samples from early breast cancer. Comparing 130 HER2-positive and 147 HER2-negative breast cancers, an SRM-MS threshold of 740 amol/µg was found that best correlated with clinical HER2 status when combined with IHC/ISH. Among HER2-positive breast cancers, patients with level of HER2 higher than 2200 amol/µg were considered as superexpressors and had significant higher DFS and OS than non superexpressors when they were treated with anti-HER2 therapy. Thus, HER2 protein quantification may actually predict outcome in HER2-positive early disease treated with anti-HER2 drugs. Of note, in our study, no correlation was observed between HER2 and HR expressions, nor between HER2 expression and tumor stage. Among the three HER2-positive metastatic patients included in clinical trial PIKHER2, one had a high level of HER2 and did not progress after 30 months of follow-up, whereas the two patients with low level of HER2 progressed after 3 and 22 months. Among the six inflammatory breast cancer (IBC) patients included in BEVERLY-2 trial, one had high level of HER2 and did not relapse after 60 months of follow-up, whereas of the five patients having low levels of HER2, four relapsed with metastases and three of them died from cancer progression. None of the patients included in BC-BIO trial relapsed. Therefore, high levels of HER2 could be associated with a better prognosis in HER2-positive advanced disease treated with anti-HER2 drugs. In our cohort, all HER2 positive breast cancer patients had benefits from HER2 targeted therapy, and a higher expression of HER2, EGFR, PTEN and HER3 but lower expression of phospho-HER2 correlated with trastuzumab sensitivity.

To better identify effective predictive or prognostic biomarkers using PRM, more sensitive mass spectrometers are needed to tackle the large range of HER2 expression. This increase in sensitivity could be very important to determine lower amounts of HER2 and its phosphorylated counterpart, which are at the moment under the LLOQ. These next-generation mass spectrometers should also be able to better detect and quantify low-abundant proteins such as EGFR, HER3 or PTEN, but also other downstream proteins in HER2 pathway such as PI3K or mTOR, which could potentially help predicting the efficacy of various anti-HER2 therapies [24]. The development of PRM-based approaches in FFPE tissue using microdissection [18, 19, 24] could also be a critical step in making this procedure an effective alternative that could in the future replace or complement IHC in the clinical practice. Thus, we could envision that the development of next generation mass spectrometers associated to enrichment methods as SISCAPA technology [33, 34] may make it possible the detection and quantification of pre-specified proteins in a simple blood sample, allowing dynamic therapeutic guidance over time, and perhaps a better assessment of the spatial heterogeneity of the tumors [35].

In conclusion, using PRM assay, we were able to detect and simultaneously quantify different proteins implicated in the HER2 pathway, both in BCLs and in more complex samples, such as PDXs and frozen breast cancer tissues. This approach was able to describe baseline levels of HER2 pathway proteins as well as quantitative variations of HER2 and phospho-HER2 peptides under anti-HER2 treatment. All of these protein biomarkers are potentially associated with treatment sensitivity, demonstrating the potential of PRM-based targeted proteomics as a theragnostic tool in HER2-positive breast cancer. Further technological developments are required before this approach can provide a comprehensive outline of operating molecular alterations, which would enable the development of an improved therapeutic algorithm for this disease.

## MATERIALS AND METHODS

### Cell lines and culture

We selected a panel of 17 human BCLs with various expression of HER2: BT474, CAMA1 [36]; HCC202, HCC2218, MCF7, MCF10A, MDA-MB175, MDA-MB361, MDA-MB436, MDA-MB453, SKBR3, and ZR-75-30 from the American Type Culture Collection database (ATCC), SUM-185, SUM-190, SUM-206, SUM-225 [37], http://www.cancer.med.umich.edu/breast_cell/production), and MCF7-ERBB2 (a gift from O. Segatto, Rome [26]). All cell lines were derived from human carcinomas except MCF10A, which is derived from a fibrocystic disease. The BCLs were grown using culture conditions recommended by their suppliers. As previously referenced [26], BCLs had various expression of HER2 using ICC [26–28]: negative for MCF7, MCF10A, CAMA-1, and MDA-MB436; equivocal for MCF7-ERBB2, MDA-MB453, and SUM185; and positive for SUM190, SUM206, SUM225, BT-474, HCC-202, HCC-2218, MDA-MB361, MDA-MB175, SKBR3, and ZR-75-30.

### Patients derived xenografts (PDXs)

Eight breast cancer PDXs were selected among our institutional collection [38] according to their IHC HER2 expression level: four of them being HER2-negative and four being HER2-positive (100% of cells for three PDXs and only 1% of cells for one PDX, which could be considered as negative) (Figure 6). PDXs were lysed using lysis buffer with Triton 1%, and one protease and phosphatase inhibitors tab (Thermo Fisher Scientific^®^). Tumors were then crushed in a multicrystaller (3 cycles of 2 minutes, 20 Hertz) before being disposed on a wheel at 4° C overnight.

### Breast cancer frozen tissues

Forty-six breast cancer frozen tissues were analyzed to determine different profiles of protein expression (for the tumors characteristics see Supplementary Table 1).

Twenty-six samples had been collected from patients treated at Institut Paoli-Calmettes at various stages of the disease: four from HER2-positive advanced breast cancer patients enrolled in the PIKHER2 study (NCT01589861) [39], six from HER2-positive IBC patients enrolled in the BEVERLY-2 trial -(NCT00717405) [40], sixteen from HER2-positive and negative early breast cancer patients enrolled in the prospective institutional BC-BIO cohort (NCT01521676). All patients given informed consent. The ethics committee « Comité de Protection des Personnes Sud Méditerranée I, Institut Paoli Calmettes, Marseille, France » approved the studies: BEVERLY-2 the March 29, 2009; BC-BIO the Nov 17, 2011; and PIKHER2 the December 14, 2012. Breast cancer samples were lysed and proteins extracted using the same process as PDXs.

In addition, twenty protein lysates extracted from early breast cancer samples as already described [41] were obtained from the Biological Resource Center of the Montpellier Cancer Institute (IRCM) (Biobank number BB-0033-00059), France. Considering these samples, this study was reviewed and approved by the Montpellier Cancer Institute Institutional Review Board (ID number ICM-CORT-2016-02).

### Cell proliferation measurements

To determine the dose level for *in vitro* trastuzumab and/or lapatinib treatments, exponentially growing SKBR3 cells were harvested and plated on 96-well plates at 5000 cells/well. After 24 hours, trastuzumab was dissolved in sterile water at 21 mg/mL and added to the culture at concentrations of 1, 5, 10, 50, 100, and 1000 μg/mL. Lapatinib (Selleck Chem^®^) was added to the culture at concentrations of 0.1, 0.25, 0.5, 1, 5, 10, and 100 μmol, with 0.5% DMSO (Diméthylsulfoxide). Fresh medium lacking trastuzumab and/or lapatinib was added to control wells. After 72 h incubation, the number of viable cells was measured by using the Cell Titer-Glo luminescent cell viability assay (Promega corporation^®^). This number was expressed as the percentage of viable cells in treated condition compared to untreated cells. For trastuzumab, a maximal effect was obtained at 10 μg/mL. For lapatinib, EC50 (Effective concentration, corresponding to the concentration which induces a response halfway between baseline and maximal effect: I.D 50% of cell death was observed) was determined for each BCL. All experiments were done in triplicate and independently replicated at least twice.

### Western blot

Western blot analyses were done using NuPAGE Bis-Tris Gels (Thermo Fisher Scientific^®^) and MOPS SDS running buffer. Membranes were saturated 30 minutes at room temperature (RT) with TBS-T (Tris buffer saline with Tween) 0.05% + BSA (Bovine serum albumin) 5%, then incubated overnight à +4° C under gentle agitation with specific primary antibodies. Membranes were then washed three times with TBS-T buffer and incubated under gentle agitation with secondary antibody one hour at RT. Membranes were washed three more times and proteins recognized by the antibody were revealed by enhanced chemiluminescence kit (34076; Thermo Fisher Scientific^®^) on LAS3000 FUJI with incremental measure mode. Antibodies used were anti HER2 (2165), Phospho-HER2 (9923), EGFR (2232), and PTEN (9583) from Cell Signaling^®^ with 1:1000 dilution. The control antibody was a monoclonal anti-β-Actin with 1:5000 dilution (Sigma-Aldrich^®^).

### Mass spectrometry

#### Sample preparation

Proteins were quantified using Bradford protein assay (Bio-Rad^®^). Proteins (80 µg) were deposited in the wells of Bolt 4–12% Bis-Tris Plus Gels (Thermo Fisher Scientific^®^) and predetermined areas corresponding to the proteins of interest were cut using a GridCutters (Multiple gel band excision from polyacrylamide gel; Gel Company, Life Sciences^®^). Each sample band was reduced by TCEP 50 mM (60 min, 60° C), alkylated by iodoacetamide 84 mM (30 min, RT) and digested overnight by adding trypsin (Promega^®^). Samples were dried under vacuum and stored at −20° C. AQUA heavy peptides (Thermo Fisher Scientific^®^) with the same physico-chemical properties were added to the sample before injection to the mass spectrometer.

#### Reversed-phase liquid chromatography on the Q-Exactive

Peptides were analyzed on the Q-Exactive instrument (Thermo Scientific^®^) connected to an Ultimate 3000 Rapid Separation LC (Dionex^®^). Fractions were loaded onto the enrichment column (C18 PepMap100, 100 μm ID, 100 Å pore size, 5 μm particle size, Dionex^®^) using 2% ACN (acetonitrile), 0.1% FA (formic acid). After the analytical column (C18 PepMap100, 75 μm ID, 100 Å pore size, 2 μm particle size) was switched in-line, the nano pump delivered a 64 min linear gradient from 2.5% ACN, 0.1% FA to 44% ACN, 0.1% FA at 300 nL/min flow rate. Instrument method for the high resolution Q-Exactive mass spectrometer (Thermo Scientific^®^) was set up in data dependent mode to switch consistently between MS and MS/MS. Peptide fragments ions were measured in a survey full scan acquired in the Orbitrap in the range of m/z 300–1,700 at a FWHM resolution of 35,000 at 200 m/z. The pre-determined precursor ions were selected and HCD (Higher-energy collisional dissociation) fragmentation was performed at specific collision energy. Fragment ions were ejected from the HCD cell and read out in the Orbitrap mass analyzer at a FWHM resolution of 35,000. Raw files generated from mass spectrometry analysis were processed with Proteome Discoverer 1.3 (Thermo fisher Scientific^®^). This software was used to search data and create a spectral library via Sequest and Mascot servers against the Human database subset of the Uniprot database (version 2013.06) containing 122 191 entries. Database searches were done using the following settings: trypsin enzyme as cleavage and N- terminal acetylation as variable modification. Mass tolerance of 6 ppm and 0.1 Da were used respectively for precursors and fragment ions during search analysis.

### Protein identification and quantification

LC-MSMS acquisitions were analyzed using Skyline software (version 3.7) (http://proteome.gs.washington.edu/software/skyline) [42] for quantification. The peptide setting was set to Trypsin as cleavage specificity, using none missed cleavages. All peptides containing Methionine or Cysteine were excluded. The spectral library provides a quick and accurate procedure to match experimental MS/MS spectra with the collection available in the library yielding a dot product probability (dotp). The spectra filter options were set on two and three charge states for precursors, and the six most intense “b“ and/or “y” fragments were selected for the MS/MS. MS/MS spectra filtering was set up at a resolving power of 140000 (at 200 m/z) and Orbitrap as mass analyzer. MS/MS spectra peak picking/integration and the identification/quantification of the precursors were assessed by matching with the spectral library. In addition, confident peak integration corresponding to the extracted ion chromatogram of the six most intense fragments of a selected precursor was adjusted manually to avoid potential interferences.

### Statistical analyses

Data plot columns and the *t* test calculations were done using GraphPad Prism version 5.00 for Windows (GraphPad Software^®^, San Diego, USA, www.graphpad.com). We use an alpha level of 0.05 for all statistical tests. On graphics, *p*-value is reported using the following schema: ns = non significant; ^*^p < 0.05; ^**^p < 0.01; ^***^p < 0.001. HeatMap and PCA analysis were done using Perseus software version 1.5.1.6 [43].

## SUPPLEMENTARY MATERIALS FIGURES AND TABLES





## Abbreviations

ACN: acetonitrile
BCL: Breast cancer cell line
BSA: Bovine serum albumin
CV: coefficient of variations
DFS: Disease free survival
DMSO: Diméthylsulfoxide
EC: Effective concentration
EGFR: Epidermal Growth Factor Receptor, also named HER1
FA: formaldehyde acid
FFPE: Formalin-fixed, paraffin-embedded
FISH: Fluorescent *in situ* hybridization
HCD: Higher-energy collisional dissociation
HER: Human Epidermal Growth Factor Receptor
HR: Hormone Receptor
ICC: Immunocytochemistry
IHC: Immunohistochemistry
LC-SRM: Liquid Chromatography-Selected Reaction Monitoring
LLOD: Lower limit of detection
LLOQ: Lower limit of quantification
MOPS: 3-(N-morpholino) propanesulfonic acid buffer
OS: Overall survival
PCA: Principal component analysis
PDX: Patient-derived xenografts
PRM: Parallel Reaction monitoring
PTEN: Phosphatase and tensin homolog
RT: Room temperature
SDS: sodium dodecyl sulfate buffer
SE: Standard error
SIS: stable isotopic peptides
SRM: Selected reaction monitoring
SSP: single sample predictor classification
TBS-T: Mixture of Tris buffer saline with Tween (Polysorbate 20)
TCEP: Tris (2-carboxyethyl) phosphine
