# Supplementary material for: Development of parallel reaction monitoring (PRM)-based quantitative proteomics applied to HER2-Positive breast cancer
Source: Oncotarget. 2018 Sep 18;9(73):33762–77. doi: 10.18632/oncotarget.26031 (PMC6173470; doi:10.18632/oncotarget.26031)
Supplement: Supplementary file 1 [file oncotarget-09-33762-s001.pdf]

Development of parallel reaction monitoring (PRM)-based quantitative proteomics applied to HER2-Positive breast cancer

SUPPLEMENTARY MATERIALS

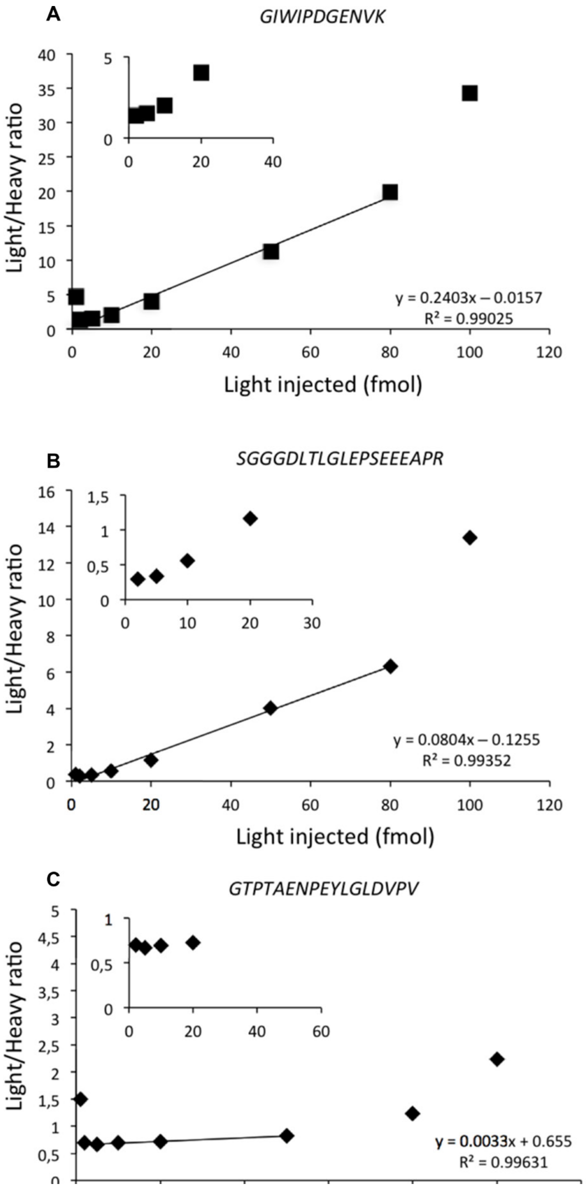

Supplementary Figure 1: Calibration curve other HER2 proteotypic peptides (**A:** GIWIPDGENVK; **B:** SGGGDLTLGLEPSEEEAPR; **C:** GTPTAENPEYLGLDVPV). The calibration curve was done in a pooled matrix of 17 BCL. Selective bands not containing protein of interest was cut, and trypsin digested. Heavy synthetic peptide was added before injection à the concentration of 10 fmol in each sample. Light synthetic peptide was added at increasing concentrations from 1 fmol to 100 fmol. Abciss represents the quantity of light synthetic peptide injected; vertical axis represents the light/heavy ratio obtained using PRM. Data are represented as mean of six technical replicates.

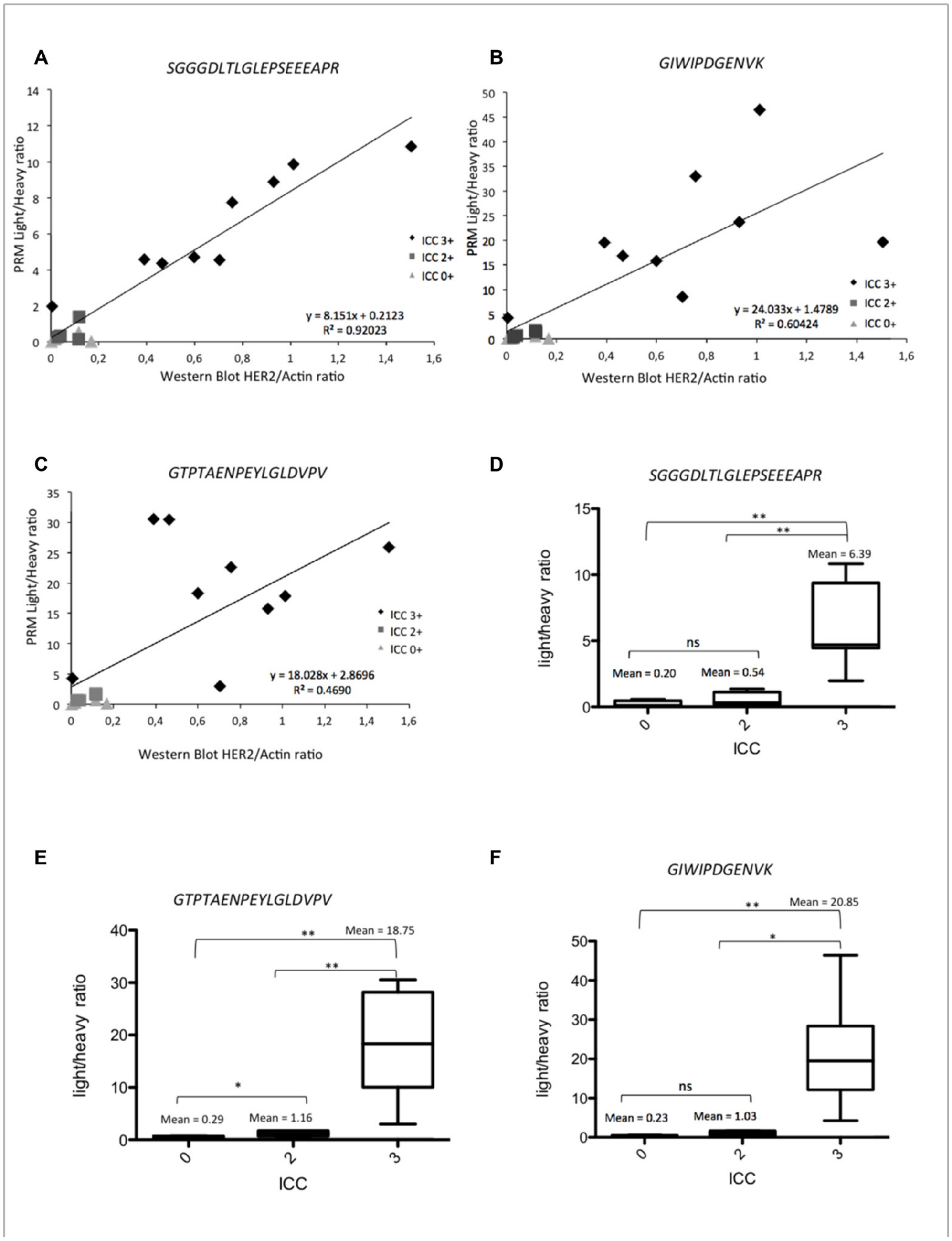

Supplementary Figure 2: Correlation between Light/Heavy ratio obtained using PRM and Western Blot and the gold standard ICC for HER2. Analysis of 17 BCLs. (**A**–**C**) Horizontal axis represents the HER2/actin ratio obtained with western blot. Vertical axis is the light/heavy ratio of the HER2 peptides (GIWIPDGENVK; SGGGDLTLGLEPSEEEAPR; GTPTAENPEYLGLDVPV) of the corresponding BCL. We also represented ICC classification of these BCL (light grey: none expression of HER2, dark grey: equivocal in ICC; black: overexpression of HER2 in ICC). (**D**–**F**) Box plot representing the light/heavy ratio of GIWIPDGENVK, SGGGDLTLGLEPSEEEAPR, and GTPTAENPEYLGLDVPV peptide depending on ICC status. ns = non significant; $^{*}p < 0.05$; $^{**}p < 0.01$; $^{***}p < 0.001$. Data are represented as mean of six technical replicates ± standard deviation.

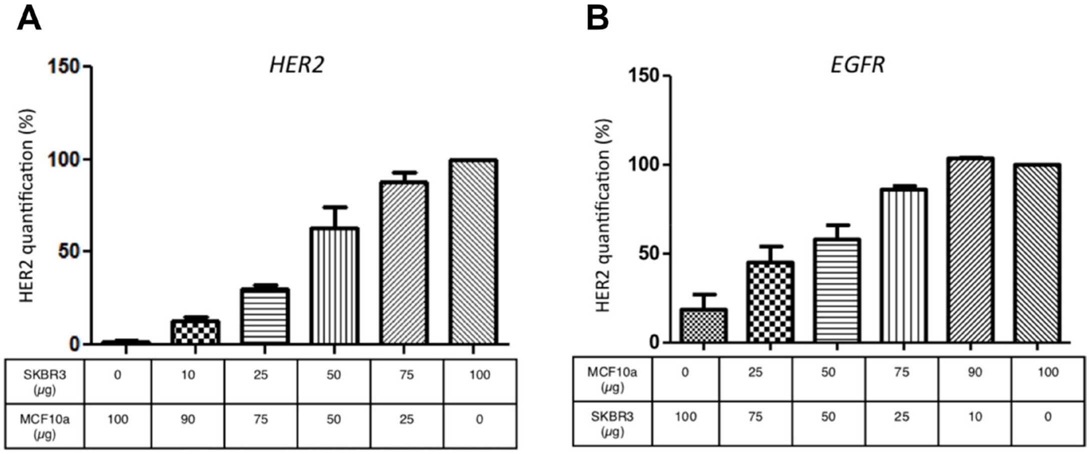

Supplementary Figure 3: Represents various quantity of SKBR3 and MCF10a for a total amount of proteins of 100 µg in each breast cancer cell line sample and the corresponding quantification of HER2 (**A**) and EGFR (**B**) measured using PRM, expressed in percent.

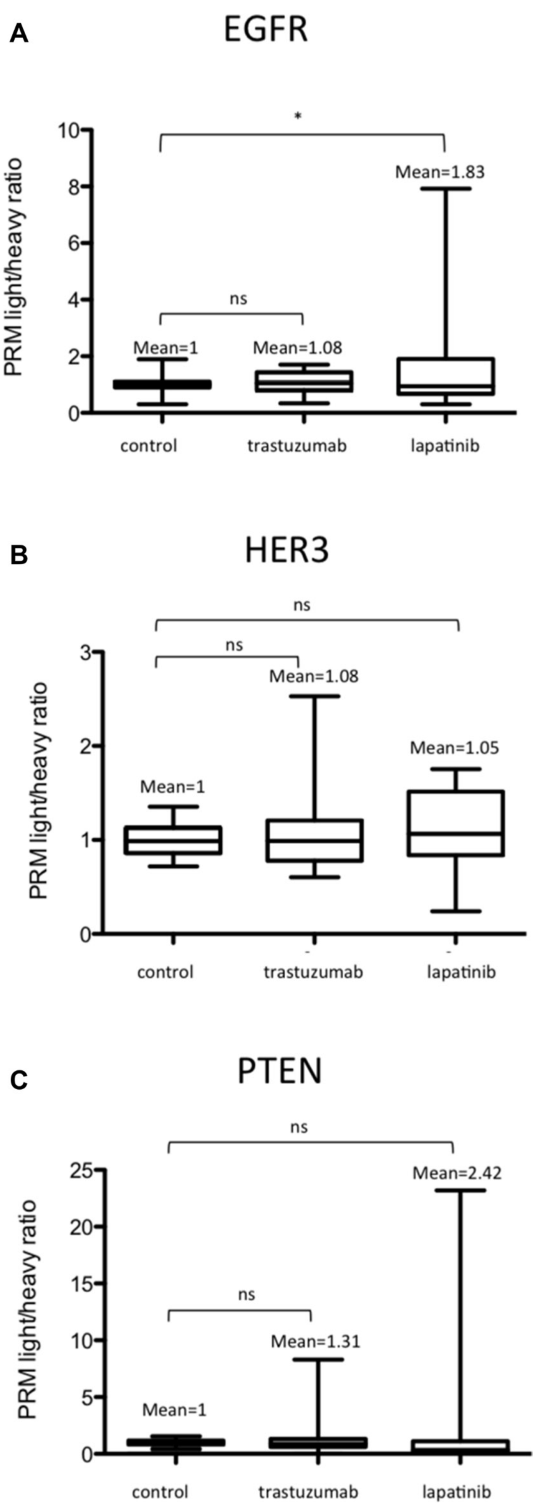

Supplementary Figure 4: Represents the relative quantification of EGFR (**A**), HER3 (**B**) and PTEN (**C**) peptides on the five BCLs (BT474, SKBR3, SUM190, SUM225 and ZR75-30) in control condition and under trastuzumab or lapatinib treatment. Each plot represents the mean of the two proteotypic peptides for each protein (GVWIPEGESIK and LAEVPDLLEK for HER3; GSTAENAEYLR and IPLENLQIIR for EGFR ; YFSPNFK; GVTIPSQR for PTEN). Data normalized versus control are represented as mean of six technical replicates ± SEM.

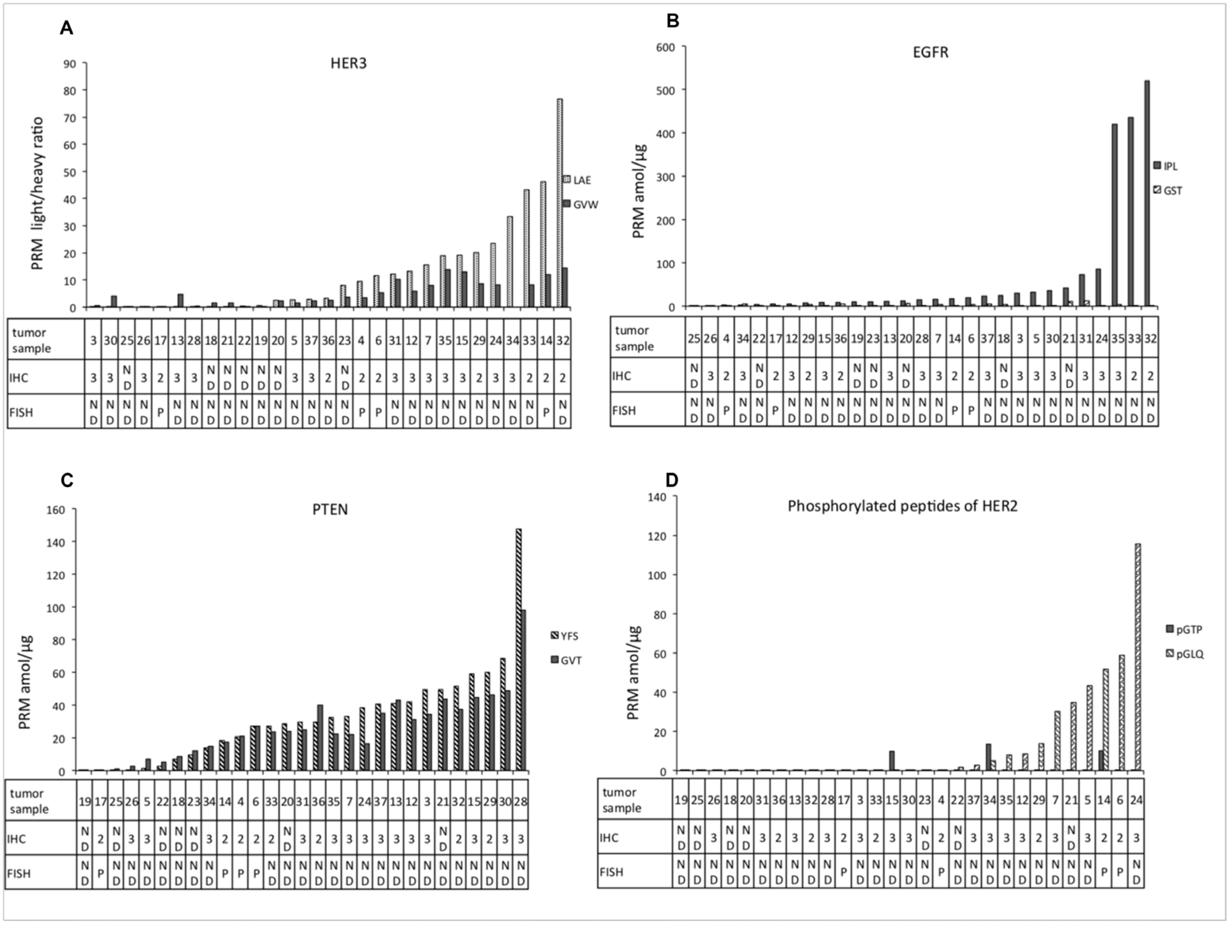

Supplementary Figure 5: Represents the quantification of other HER2 proteins pathway in PRM for the HER2 positive breast tumors. (**A**) 2 HER3 proteotypic peptides (GVWIPEGESIK; LAEVPDLLEK), relative quantification; (**B**) 2 EGFR proteotypic peptides (GSTAENAEYLR; IPLENLQIIR) absolute quantification; (**C**) 2 PTEN proteotypic peptides (YFSPNFK; GVTIPSQR), absolute quantifcation; (**D**) 2 phospho-HER2 peptides (GLQSLPTHDPSPLQR; GTPTAENPEYLGLDVPV), absolute quantification. Data are represented as mean of four replicates ± standard deviation.

Supplementary Table 1: Histologic characteristics of the 46 breast cancer frozen tissues

TUMOUR	TRIAL	T	N	M	HER2	Hormone receptors	KI67	Grade SBR
1	BC-BIO	1A	0	0	NEGATIVE	POSITIVE	1%	II
2	BC-BIO	2	1mi	0	NEGATIVE	POSITIVE	35%	II
3	BC-BIO	1C	1A	0	POSITIVE	NEGATIVE	50%	III
4	BC-BIO	2	3A	1	POSITIVE	POSITIVE	30%	III
5	BC-BIO	1B	0	0	POSITIVE	POSITIVE	2%	III
6	BC-BIO	1C	0	0	POSITIVE	POSITIVE	70%	III
7	BC-BIO	2	1A	0	POSITIVE	POSITIVE	50%	III
8	BC-BIO	1C	1A	0	NEGATIVE	NEGATIVE	40%	III
10	BC-BIO	1C	0	0	NEGATIVE	POSITIVE	25%	II
11	BC-BIO	1C	0	0	NEGATIVE	POSITIVE	60%	III
12	BC-BIO	1C	0	0	POSITIVE	NEGATIVE	30%	II
13	BC-BIO	1C	0	0	POSITIVE	POSITIVE	15%	II
14	BC-BIO	1C	0	0	POSITIVE	NEGATIVE	80%	III
15	BC-BIO	2	1A	0	POSITIVE	POSITIVE	50%	III
16	BC-BIO	1C	0	0	NEGATIVE	POSITIVE	80%	III
17	BC-BIO	1C	0	0	POSITIVE	POSITIVE	30%	II
18	BEVERLY II	4D	x	0	POSITIVE	NEGATIVE	ND	II
19	BEVERLY II	4D	x	0	POSITIVE	NEGATIVE	ND	ND
20	BEVERLY II	4D	x	0	POSITIVE	POSITIVE	ND	III
21	BEVERLY II	4D	x	0	POSITIVE	NEGATIVE	ND	III
22	BEVERLY II	4D	x	0	POSITIVE	NEGATIVE	ND	III
23	BEVERLY II	4D	x	0	POSITIVE	NEGATIVE	ND	III
24	PIK-HER2	ND	ND	ND*	POSITIVE	POSITIVE	ND	III
25	PIK-HER2	ND	ND	ND*	POSITIVE	POSITIVE	ND	II
26	PIK-HER2	ND	ND	ND*	POSITIVE	NEGATIVE	ND	III
27	PIK-HER2	ND	ND	ND*	NEGATIVE	POSITIVE	15%	III
28	montpellier	2	1	0	POSITIVE	POSITIVE	ND	III
29	montpellier	2	0	0	POSITIVE	POSITIVE	ND	III
30	montpellier	2	1	0	POSITIVE	POSITIVE	ND	II
31	montpellier	1	0	0	POSITIVE	NEGATIVE	ND	II
32	montpellier	2	0	0	POSITIVE	POSITIVE	ND	III
33	montpellier	2	0	0	POSITIVE	POSITIVE	ND	III
34	montpellier	1	1	0	POSITIVE	NEGATIVE	ND	III
35	montpellier	2	1	0	POSITIVE	POSITIVE	ND	II
36	montpellier	1	1	0	POSITIVE	POSITIVE	ND	II
37	montpellier	2	0	0	POSITIVE	POSITIVE	ND	III
38	montpellier	2	0	0	NEGATIVE	POSITIVE	ND	III
39	montpellier	2	1	0	NEGATIVE	POSITIVE	ND	III
40	montpellier	2	0	0	NEGATIVE	POSITIVE	ND	III
41	montpellier	2	0	0	NEGATIVE	POSITIVE	ND	I
42	montpellier	2	0	0	NEGATIVE	POSITIVE	ND	II
43	montpellier	2	0	0	NEGATIVE	POSITIVE	ND	II
44	montpellier	2	0	0	NEGATIVE	NEGATIVE	ND	III
45	montpellier	3	1	0	NEGATIVE	POSITIVE	ND	III
46	montpellier	3	0	0	NEGATIVE	POSITIVE	ND	II
47	montpellier	2	0	0	NEGATIVE	POSITIVE	ND	II

*All PIKHER2 patients had biospy from metastatic relapse.

Supplementary Table 2: Absolute quantification of HER2, EGFR, and PTEN proteins, and two HER2 phospho-peptides, relative quantification of HER3; mean and standard-error (SE) for all breast cell lines (BCLs). See Supplementary_Table_2
