# Supplementary material for: Development of parallel reaction monitoring (PRM)-based quantitative proteomics applied to HER2-Positive breast cancer
Source: Oncotarget. 2018 Sep 18;9(73):33762–77. doi: 10.18632/oncotarget.26031 (PMC6173470; doi:10.18632/oncotarget.26031)
Supplement: Supplementary file 2 [file oncotarget-09-33762-s002.pdf]

Table 2: absolute quantification of HER2, EGFR, and PTEN proteins, and two HER2 phospho-peptides, relative quantification of HER3 ; mean and standard-error (SE) for all breast cell lines (BCLs) .

	HER2								pHER2				EGFR				HER3				PTEN			
BCL	mean GIWI	SE GIWI	mean GLQ	SE GLQ	mean SGG	SE SGG	mean GTP	SE GTP	mean pGLQ	SE pGLQ	mean pGTP	SE pGTP	mean IPL	SE IPL	mean GST	SE GST	mean GVW	SE GVW	mean LAE	SE LAE	mean GVT	SE GVT	mean YFS	SE YFS
BT474	2220.15	311.59	1865.51	737.30	785.71	545.32	1630.81	1375.05	45.78	40.74	133.56	192.70	<LOQ	28.89	49.23	61.35	5.90	4.09	0.03	0.02	<LOQ	5.87	<LOQ	10.41
CAMA-1	<LOQ	48.82	<LOQ	72.94	115.12	67.70	<LOQ	62.64	34.56	65.96	<LOQ	0.40	<LOQ	ND	<LOQ	33.89	0.86	0.51	0.02	0.04	<LOQ	3.98	<LOQ	2.37
HCC202	3817.35	319.79	4131.07	554.39	1146.68	131.47	4892.57	1004.27	86.95	14.81	<LOQ	2.05	294.03	145.77	240.14	98.21	8.61	0.99	0.05	0.01	49.78	12.86	<LOQ	17.62
HCC2218	12129.60	1658.89	9859.44	3189.77	2824.80	805.99	6338.46	4210.55	207.62	86.80	<LOQ	15.47	68.97	31.32	62.23	65.37	17.65	11.02	0.28	0.40	<LOQ	14.35	<LOQ	15.27
MCF10A	<LOQ	7.31	<LOQ	5.31	<LOQ	2.81	<LOQ	5.74	<LOQ	1.70	<LOQ	0.11	270.23	107.55	110.65	57.97	0.04	0.02	0.00	0.00	<LOQ	16.06	<LOQ	26.68
MCF7	<LOQ	11.64	<LOQ	29.97	<LOQ	14.28	<LOQ	44.49	<LOQ	3.54	<LOQ	0.03	<LOQ	2.34	<LOQ	0.77	0.35	0.11	0.00	0.00	71.03	25.54	78.98	54.88
MCF7-E2	<LOQ	84.86	429.18	59.45	371.25	46.56	425.21	174.76	<LOQ	0.62	17.63	8.54	<LOQ	2.81	<LOQ	24.81	2.79	0.35	0.00	0.00	119.01	41.10	127.40	64.20
MDA MB175	<LOQ	206.04	429.94	100.02	<LOQ	14.15	438.34	102.17	<LOQ	6.15	115.65	ND	<LOQ	59.25	34.83	23.82	0.22	0.11	0.00	0.00	57.69	9.27	<LOQ	7.71
MDA MB361	<LOQ	265.44	977.96	222.89	530.21	87.75	1100.64	115.21	<LOQ	8.20	28.91	10.51	<LOQ	20.34	38.99	23.87	3.98	0.66	0.01	0.00	<LOQ	11.66	<LOQ	23.18
MDA MB436	<LOQ	9.75	<LOQ	6.96	<LOQ	3.23	<LOQ	10.76	<LOQ	14.30	<LOQ	0.68	225.84	25.23	227.24	33.45	0.08	0.02	0.01	0.01	<LOQ	2.20	<LOQ	1.72
MDA MB453	<LOQ	94.26	<LOQ	46.90	69.08	34.92	139.52	38.28	<LOQ	1.95	<LOQ	2.79	<LOQ	2.01	<LOQ	7.00	0.52	0.26	0.00	0.00	<LOQ	2.51	<LOQ	5.13
SKBR3	3771.87	2322.94	5803.35	699.04	2017.82	1155.40	6166.53	532.29	72.64	9.30	77.68	38.06	165.54	71.57	168.50	79.72	15.15	8.68	0.04	0.01	44.21	5.79	<LOQ	10.86
SUM185	<LOQ	32.69	<LOQ	20.78	56.57	21.91	<LOQ	24.68	<LOQ	10.20	<LOQ	7.13	110.77	45.57	109.30	24.48	0.42	0.16	0.01	0.01	171.15	17.97	187.74	20.41
SUM190	3424.49	1486.19	6329.02	2321.82	1056.12	434.66	7733.62	2321.90	166.48	49.15	467.10	80.18	<LOQ	31.80	<LOQ	4.38	7.93	3.26	0.10	0.03	<LOQ	16.85	<LOQ	25.48
SUM206	3669.47	3318.60	5755.47	2712.11	873.03	743.18	7164.41	3417.28	176.70	83.24	534.84	146.57	<LOQ	24.42	33.60	31.73	6.56	5.58	0.10	0.05	63.50	7.17	72.36	20.92
SUM225	5105.40	1441.87	8766.52	5171.32	2289.49	192.45	5423.11	2300.38	152.18	94.87	1202.10	631.96	<LOQ	4.31	75.88	89.22	17.19	1.45	0.09	0.06	<LOQ	7.57	<LOQ	16.48
ZR75-30	8795.80	3499.14	7819.16	2043.82	1798.92	543.53	6140.64	1336.34	174.92	37.25	49.47	19.64	<LOQ	ND	<LOQ	ND	13.51	4.08	0.10	0.02	100.41	27.06	100.32	59.31

ABBREVIATIONS: *GIWI : GIWIPDGENVK ; GLQ : GLQSLPTHDPSPLQR ; SGG : SGGGDLTLGLEPSEEEAPR ; GTP : GTPTAENPEYLGLDVPV; pGLQ : GLQSLPTHDP**S(1107)**PLQR ; pGTP : GTPTAENPE**Y(1248)**LGLDVPV ; IPL : IPLENLQIIR; GST : GSTAENAEYLR ; GVW : GVWIPEGESIK ; LAE : LAEVPDLLEK ; GVT : GVTIPSQR ; YFS : YFSPNFK.*
